# Adhesion molecules in peritoneal dissemination: function, prognostic relevance and therapeutic options

**DOI:** 10.1007/s10585-016-9791-0

**Published:** 2016-04-13

**Authors:** Nina Sluiter, Erienne de Cuba, Riom Kwakman, Geert Kazemier, Gerrit Meijer, Elisabeth Atie te Velde

**Affiliations:** Department of Surgery, VU University Medical Centre, De Boelelaan 1117, 1081 HV Amsterdam, The Netherlands; Department of Pathology, VU University Medical Centre, De Boelelaan 1117, 1081 HV Amsterdam, The Netherlands; Department of Surgical Oncology, VU University Medical Centre, De Boelelaan 1117, 1081 HV Amsterdam, The Netherlands; Department of Pathology, Antoni van Leeuwenhoek Hospital (NKI-AVL), Plesmanlaan 121, 1066 CX Amsterdam, The Netherlands

**Keywords:** Peritoneal metastases, Colorectal cancer, Hipec, Adhesion, Predictive biomarkers, Prognosis

## Abstract

Peritoneal dissemination is diagnosed in 10–25 % of colorectal cancer patients. Selected patients are treated with cytoreductive surgery and hyperthermic intraperitoneal chemotherapy. For these patients, earlier diagnosis, optimised selection criteria and a personalised approach are warranted. Biomarkers could play a crucial role here. However, little is known about possible candidates. Considering tumour cell adhesion as a key step in peritoneal dissemination, we aim to provide an overview of the functional importance of adhesion molecules in peritoneal dissemination and discuss the prognostic, diagnostic and therapeutic options of these candidate biomarkers. A systematic literature search was conducted according to the PRISMA guidelines. In 132 in vitro, ex vivo and in vivo studies published between 1995 and 2013, we identified twelve possibly relevant adhesion molecules in various cancers that disseminate peritoneally. The most studied molecules in tumour cell adhesion are integrin α2β1, CD44 s and MUC16. Furthermore, L1CAM, EpCAM, MUC1, sLe^x^ and Le^x^, chemokine receptors, Betaig-H3 and uPAR might be of clinical importance. ICAM1 was found to be less relevant in tumour cell adhesion in the context of peritoneal metastases. Based on currently available data, sLe^a^ and MUC16 are the most promising prognostic biomarkers for colorectal peritoneal metastases that may help improve patient selection. Different adhesion molecules appear expressed in haematogenous and transcoelomic spread, indicating two different attachment processes. However, our extensive assessment of available literature reveals that knowledge on metastasis-specific genes and their possible candidates is far from complete.

## Introduction

Colorectal cancer (CRC) is the third most common cancer worldwide [1]. Approximately half of CRC patients develop distant metastasis, mainly through haematogenous dissemination to the liver [2, 3]. 10–25 % of CRC patients eventually develop peritoneal metastases (PM) [3, 4] and in up to 25 % of these patients the peritoneum is the only site of metastasis [4, 5]. Typically, untreated PM are associated with poor survival rates, even when treated with modern systemic chemotherapy [6–8].

Macroscopic complete cytoreductive surgery (CRS) combined with hyperthermic intraperitoneal chemotherapy (HIPEC) is the preferred therapeutic strategy for patients with isolated PM [9, 10], resulting in a 5 year survival rate equal to that of patients undergoing resection for colorectal liver metastases (35–45 %) [11, 12] and a median survival of 33 months [6, 13, 14].

Despite the success of CRS and HIPEC, this treatment has morbidity and mortality rates of 15–34 and 5 % respectively [5, 6, 11, 15]. Therefore, selection of those patients that will benefit most from this treatment is of utmost importance. Other challenges in this field are earlier diagnosis and a more personalised approach, indicating that the choice of treatment should depend on a cancer’s specific biology instead of a ‘one size fits all’ approach [16]. Based on the hypothesis that the clinical behaviour of PM in CRC is dictated by biological mechanisms, read-outs of biological information (i.e., biomarkers) are very promising aids in addressing these clinical needs.

More specifically, understanding molecular mechanisms entails knowledge on molecules contributing to peritoneal dissemination. Peritoneal dissemination is considered to be a multistep process in which tumour cells must detach from their primary tumour, gain motility and evade anoikis. Once a viable, free cancer cell is present in the peritoneal cavity, adherence to the peritoneal surface is required in order to ultimately invade the peritoneum, proliferate and form PM [16].

Accordingly, the presence of free-floating cancer cells in the peritoneal cavity is known to increase the risk of peritoneal dissemination [9, 17–20]. Hence, exfoliation of cancer cells into the peritoneal cavity might lead to PM formation in patients presenting with CRC growing through the serosa (T4 stage) [9, 21, 22]. Also patients undergoing abdominal surgery have an increased risk of PM formation, possibly through the combination of surgery-induced tumour spill and upregulation of adhesion molecules due to post-operative inflammation [9, 20, 23]. Thus, in several groups of patients, tumour cell adhesion to the peritoneum appears to be pivotal in peritoneal dissemination. Molecules responsible for adhesion might therefore be promising biomarkers that can be used in diagnosis, prognosis and therapy of PM. Considering tumour cell adhesion as a key step in the formation of PM [16, 24], we aimed to provide an overview of the functional importance of several attachment markers and to subsequently evaluate their roles in diagnosis, prognosis and therapy.

## Methods

### Literature search

A systematic literature search was conducted using the PubMed database of the U.S. National library of Medicine (medline and pre-medline). Table 1 shows the breakdown of search terms and Boolean combinations.

**Table 1:** Search strategy

Cancer types		Peritoneal metastases		Adhesion molecules	
Cancer		*AND*		*AND*	
Carcinoma	*AND*	Peritoneal		Attachment	
	Colorectal	Peritoneum		Adherence	
	Colon	Mesothelium	*AND*	Adhesion	*AND*
	Rectal		Metastasis^a^		Molecule^a^
	Gastric		Peritoneal carcinomatosis		Cell adhesion molecules^a^
	Pancreas		Pseudomyxoma Peritonei^a^		Cell adhesion^a^
	Pancreatic		Peritoneal neoplasms/secondary^a^		
	Pseudomyxoma peritonei				
*OR*					
Colorectal neoplasms^a^					
Stomach neoplasms^a^					
Pancreatic neoplasms^a^					
Ovarian neoplasms^a^					

^a^Mesh term

### Inclusion- and exclusion criteria

All full-text papers, in English, published between January 1995 and January 2013 were considered in order to identify as many important adhesion molecules as possible. For this purpose, in vitro, ex vivo and in vivo studies on PM from colorectal, ovarian, gastric and pancreatic cancer as well as pseudomyxoma peritonei (PMP) were assessed. These types of cancer all disseminate to the peritoneum and can be treated with CRS and HIPEC. Literature on PM from CRC is scarce. As such, literature on other malignancies disseminating to the peritoneum may contain important information. Irrespective of the specific epithelial malignancy, cancer cells disseminate to the peritoneum theoretically following the same stepwise process [16]. Although the first steps, i.e. detachment from the primary tumour, gaining motility and evading anoikis, might differ between these cancers in respect to several molecules, cancer cells of these types of cancer have to attach to the peritoneal surface to form a peritoneal deposit [16]. Accordingly, the same molecular mechanisms might be important in these cancers and the same interventions might be useful in preventing peritoneal dissemination. No reviews and case-reports were included. Other papers were incorporated by manually cross-referencing from publications retrieved in the initial search.

**Fig. 1:**
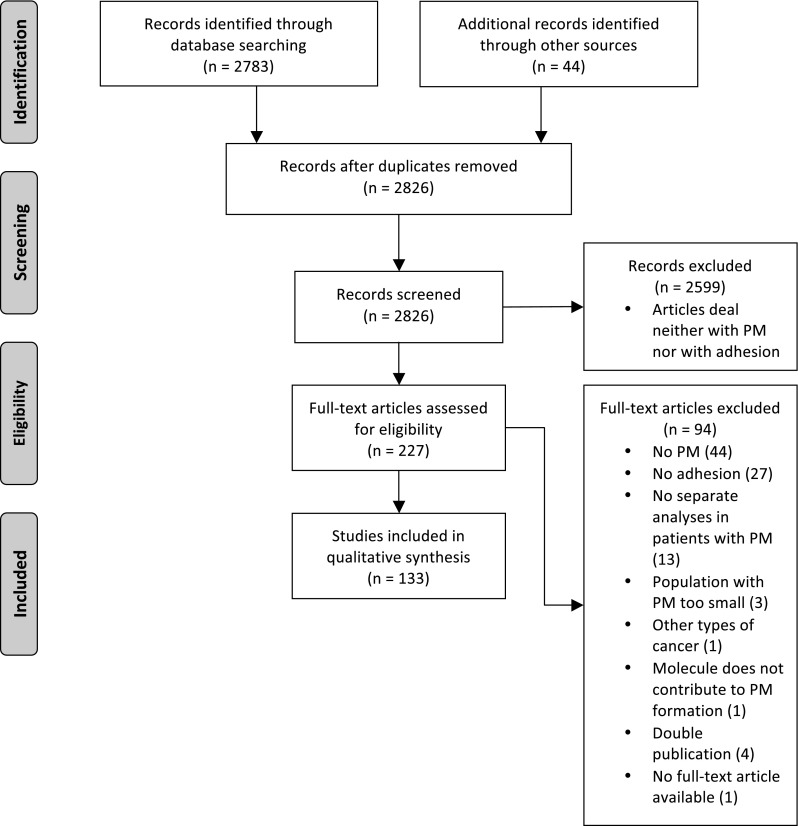
PRISMA flow chart for inclusion of the studies [152]

An additional review was conducted when deemed necessary. When studies overlapped or were duplicated, the articles with the most complete data on tumour cell adhesion to the peritoneum were retained. Figure 1 depicts the literature search and the selection process.

## Results

**Fig. 2:**
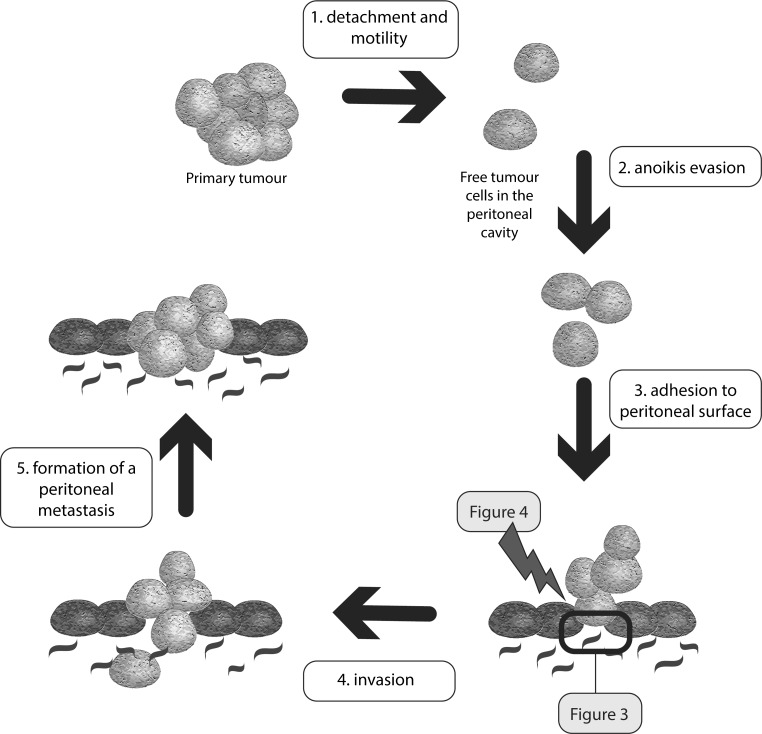
An overview of the essential steps in peritoneal dissemination. The exact molecular mechanisms in tumour cell adhesion to the peritoneum are shown in Fig. 3. Possible therapeutic options focussing on adhesion molecules are shown in Fig. 4

**Fig. 3:**
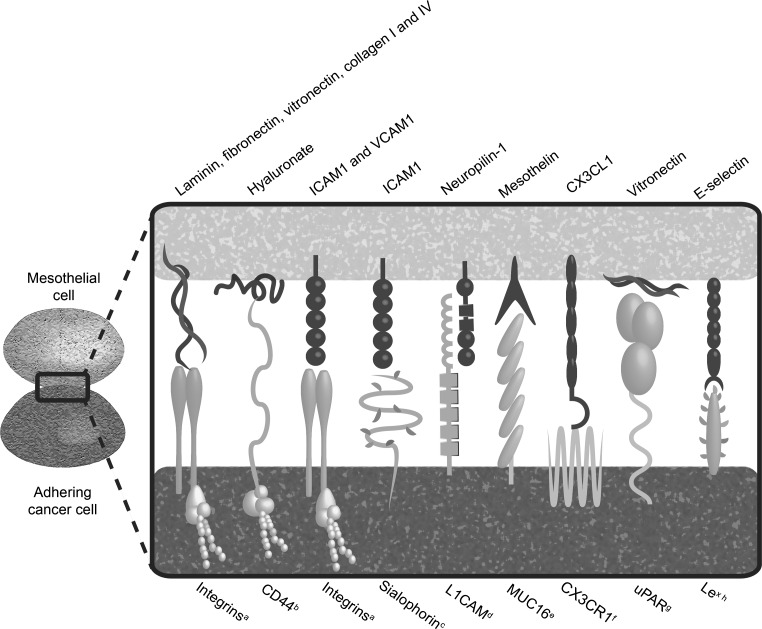
Adhesive interactions mediating tumour cell adhesion to the peritoneum. *a* Especially α2β1 expressed on colorectal [46], ovarian [26, 27, 33–42, 149], gastric [28, 31, 43–45, 150] and pancreatic [30, 47, 151] cancer cells; *b* especially CD44 and CD44 s expressed on colorectal [64], ovarian [27, 34, 65–67, 70, 76, 77, 90], gastric [28, 29, 78] and pancreatic [30] cancer cells; *c* expressed on colorectal, ovarian and pancreatic cancer cells [95]; *d* expressed on ovarian cancer cells [98–100]; *e* expressed on ovarian [40, 93, 122, 123] and pancreatic [122] cancer cells; *f* expressed on ovarian cancer cells [141]; *g* expressed on ovarian cancer cells [55]; *h* expressed on ovarian cancer cells [93, 105]

The key mechanism in PM formation is adherence of malignant cells to the peritoneal surface. Figure 2 illustrates the process of peritoneal dissemination. Figure 3 depicts the main interactions responsible for tumour cell adhesion to the peritoneum. Below, the functional and clinical importance of the adhesion molecules will be discussed.

### Integrins and integrin ligands

#### Integrins

Integrins belong to the superfamily of cell adhesion receptors. This family consists of 24 members, each of which is a heterodimer composed of α and β subunits [25]. In particular, integrin β1 [26–30] and integrin α2 [26–29, 31, 32] chains were shown to be upregulated in cancer cells with high peritoneal seeding potential. Multiple in vitro and ex vivo blocking experiments with ovarian [26, 27, 33–42], gastric [28, 31, 43–45], colon [46] and pancreatic [30, 47] cancer cells further endorse the roles of integrin α2β1 in cancer cell attachment to the peritoneum. Besides mediating adhesion of free-floating tumour cells, integrin α2β1 might also be important in the adhesion of ovarian cancer cell aggregates (i.e. spheroids) to the peritoneum, in this way promoting PM formation [40, 41].

The above-mentioned studies not only support the role of integrin α2β1 in tumour cell attachment to the peritoneum, but also suggest that integrin blocking might be a useful strategy for prevention and treatment of PM. In vivo studies suggested a role for antibodies against integrin β1 chains in prevention of colorectal [46], gastric [28, 45] and pancreatic [47] tumour cell adhesion to (traumatised) peritoneum. Furthermore, the NF-κB inhibitor dehydroxymethylepoxyquinomicin (DHMEQ) reduced expression of integrin β1 and α2 chains and was effective, both in vitro and in vivo, in preventing PM formation from gastric cancer [48]. For this purpose, other compounds that diminish integrin β1 chain expression, such as phospholipids [49], endostatin and simvastatin [42, 50] might be effective as well and are interesting to pursue further.

Although some studies describe a less prominent role for integrin β1 chains in PM formation [38, 51, 52], the majority of published literature showed the opposite. Literature on several other subunits only concerns their roles in vitro [34, 39–41, 47, 53, 54]. Their roles in vivo, therefore, remain unclear.

#### Integrin ligands

Multiple in vitro studies have indicated that the main mesothelial ligands participating in the interaction with integrins are the extracellular matrix (ECM) components vitronectin [39, 47, 52, 54, 55], fibronectin [27, 30, 34, 40, 41, 45], laminin [27, 30, 34, 40, 41, 44, 45, 56, 57] and collagen I and IV [27, 30, 34, 40, 41, 45]. Adherence of tumour cells to ECM components occurs in several ways. First, free tumour cells might enter the submesothelial compartment at places of peritoneal discontinuity, for example places that consist of milky spots [58] or places where discontinuity is induced by surgery [23, 46, 59]. Secondly, tumour cells can induce apoptosis of mesothelial cells [59]. Also, the ECM might be exposed after inflammatory mediators induce contraction of mesothelial cells and disruption of intercellular junctions [59]. These ECM components might serve as treatment targets as well, since blocking them with antibodies and peptide sequences can reduce tumour cell adhesion. For example, the fibronectin amino acid sequence RGDS and the laminin sequence YIGSR inhibited in vitro and in vivo peritoneal dissemination from gastric and ovarian cancer [26, 57, 60]. Another possible therapeutic option in gastric cancer is coupling of adriamycin to the laminin-5 peptide sequence SWKLPPS, as it increased its in vitro anticancer activity [61].

### Proteoglycans

#### CD44

The CD44 molecule is a cell-surface proteoglycan participating in cell–cell interaction, cell adhesion and cell migration [62]. In particular, CD44 isoforms originating from alternative splicing are thought to be important in tumour metastasis. The molecule is expressed on mesothelial cells and several types of cancer cells (Fig. 3). Its overexpression in gastric [29], ovarian [27] and in pancreatic [30, 63] cancer with high peritoneal seeding potential indicates a putative role for CD44 in PM formation. In vitro and ex vivo blocking experiments in several types of cancer illustrated the role of CD44 as adhesion molecule in PM formation [44, 64–70] and particularly indicated a role for the CD44 s splice variant [28, 30, 70]. Concluding from in vitro, ex vivo and in vivo studies, this molecule predominantly acts by binding to the ECM proteoglycan hyaluronan [28, 64, 65, 67, 71].

CD44 and CD44 s mediated adhesion to hyaluronan might partially be responsible for augmented cancer cell adhesion during post-operative inflammatory conditions. During this response, reactive oxygen species (ROS) [72, 73] and cytokines, for example TGF-β1, IL-1b and TNF-α [72, 74], are generated that upregulate CD44 expression and may also be responsible for the expression of other adhesion molecules [69].

Due to its suggested function in PM, CD44 s and its ligands hyaluronan are theoretically attractive therapeutic targets. In vivo blocking of CD44 s prevented PM in ovarian, gastric and pancreatic cancer [28, 30, 60, 68]. Other molecules contributing to CD44 mediated cell adhesion might also serve as therapeutic targets, e.g. urokinase plasminogen activator (uPA), multidrug resistance 1 polypeptide (MDR1) and multidrug resistance protein 2 (MRP2) [75]. A third option is inhibiting CD44 glycosylation, because this process is possibly involved in CD44 mediated adhesion [66]. The CD44 s splice variant has, despite its role in PM, an uncertain prognostic and diagnostic value [76–80].

Although—theoretically—blocking the CD44 ligand hyaluronan might prevent peritoneal dissemination, its therapeutic value is controversial: both tumour promoting and tumour repressing effects were reported after blocking CD44 intraperitoneally with hyaluronan [81–83]. Intraperitoneal application of the hyaluronan-degrading enzyme hyaluronidase, however, does yield promising in vitro results [28, 34, 64, 65]. Hyaluronidase possibly acts by degradation of mesothelial-associated hyaluronan, thereby preventing hyaluronan from interacting with CD44 on tumour cells. Another strategy is improving chemotherapeutic agent delivery to malignant cells by coupling them to hyaluronan. In vivo, promising results were seen for intraperitoneal use of hyaluronan bound cisplatin [84] and hyaluronate (ONCOFID-P) [85] bound to paclitaxel in ovarian cancer and for hyaluronan (ONCOFID-S) bound to camptothecin (SN38) in CRC [86]. Lastly, in vitro and in vivo experiments indicated a possible role for adhesion barriers, such as seprafilm and hyalurobarrier, in inhibiting peritoneal dissemination [82, 87–89].

#### Other proteoglycans

Several other proteoglycans have been described in tumour cell adhesion to the peritoneum. The proteoglycans syndecan-1, syndecan-2, syndecan-4, glypican-1 and glypican-3 were upregulated in gastric cancer with high in vitro and in vivo peritoneal seeding potential [90], suggesting a role for these molecules in peritoneal dissemination. Considering that several compounds blocking heparan sulfate and chondroitin sulfate proteoglycans, such as heparin, heparin sulfate, dermatan sulfate, chondroitin glycosaminoglycans, heparitinase, chondroitinase ABC, or methylumbelliferyl xyloside, inhibit ovarian [27, 28, 53, 90] and colorectal [91] cancer cell adhesion to ECM components, blocking these proteoglycans could be a promising therapeutic option.

### Immunoglobulin superfamily

The immunoglobulin superfamily is a large group of cell adhesion proteins, which include intercellular adhesion molecule 1 (ICAM 1), vascular cell adhesion molecule 1 (VCAM 1) and L1 cell adhesion molecule (L1CAM) [2, 92].

#### ICAM1

ICAM1 is a cell surface molecule typically expressed on endothelial cells, cells of the immune system, cancer cells [42, 69, 72, 74, 93, 94] and mesothelial cells [69, 72–74, 93, 94]. Ziprin et al. [95] demonstrated in vitro tumour cell adhesion to the peritoneum to be mediated by the interaction between mesothelial ICAM1 and CD43 (sialophorin) on colorectal, ovarian and pancreatic cancer cells. This interaction might be important under postoperative inflammatory conditions, as the inflammatory mediators TNFα [69, 72, 74, 94], IL-1α [72], IL-1β [72], IL-6 [69] and ROS [73] enhanced ICAM1 expression and stimulated PM formation. Thus, theoretically, anti-ICAM1 antibodies [42, 69] or ICAM1 downregulation with heparin [94] and simvastatin treatment [42] may be used in prevention of PM under inflammatory conditions. However, several in vitro studies on the role of ICAM1 as an adhesion molecule in PM did not show reproducible findings [42, 69, 73, 94]. Surprisingly, an in vivo study in gastric cancer even indicated that ICAM1 possibly inhibits PM formation due to ICAM1/LFA1 mediated mononuclear cell recruitment [96]. These contradictory findings make ICAM1 a dubious therapeutic target.

#### VCAM1

The membrane protein VCAM1 mediates leukocyte-endothelial cell adhesion and signal transduction [97]. The mesothelial VCAM1 is possibly responsible for tumour cell adhesion by interacting with integrin α1β1 and α4β7 on tumour cells [93]. Enhanced VCAM1 expression induced by TNF-α, ILβ [72, 74] and ROS [73] might contribute to the increased risk of PM formation after surgery. Accordingly, downregulating this molecule with anti-VCAM1 antibodies [42, 71] or simvastatin [42] might prevent peritoneal dissemination.

#### L1CAM

L1CAM is described in various processes contributing to tumour progression, such as differentiation, proliferation, migration, invasion and tumour cell adhesion [98]. Its upregulation on ovarian cancer cells with high peritoneal seeding potential indicates a role for L1CAM in PM formation. In this process, as suggested by in vitro and in vivo ovarian cancer experiments, it probably mediates adhesion to the peritoneum by interacting with mesothelial neuropilin 1 (NRP1) [99]. Although L1CAM has not yet been proven to be valuable in the prognostic and diagnostic field [100], several therapeutic strategies targeting this molecule might be promising. One option might be antibody treatment, which reduced in vivo PM formation from ovarian cancer without producing side effects [98]. Another in vivo ovarian cancer study indicated possible therapeutic relevance for radioimmunotherapy combining anti-L1CAM antibodies (chCE7 and L1-11A) with ^67^Cu-radiotherapy [101].

### Blood group antigen proteins

Several blood group antigens and related structures are expressed on tumour cells [28, 30, 102–104], including sialyl Lewis a (sLe^a^, a blood group antigen), Lewis x and sialyl Lewis x (Le^x^ and sLe^x^, two blood group antigen related structures). However, only Le^x^ [93, 105] and sLe^x^ [28, 30, 33, 106] appear to mediate tumour cell adhesion by interacting with mesothelial E-selectin [106]. Although in vitro and in vivo antibody experiments made the contribution of sLe^a^ unlikely [28, 30, 33, 102], in vivo PM formation from pancreatic cancer was inhibited after decreasing sLe^x^ and sLe^a^ biosynthesis by blocking fucosyltransferase 3 (FUT3) [107].

Despite its debatable role in tumour cell adhesion to the peritoneum, sLe^a^ detection using immunohistochemistry [104], immunocytology [103] or immunoassays in serum [108] correlated to the presence of PM, peritoneal recurrence [109, 110] and poor prognosis [103, 108–112]. In the diagnostic and prognostic field, especially serum and peritoneal lavage levels of CA19-9, a monoclonal antibody against sLe^a^, were shown to be predictive. However, due to its low sensitivity and contradictory results in patients with gastric cancer, CRC and PMP [80, 103, 104, 108, 109, 111–120], CA19-9 is not yet qualified for clinical use as a single marker. Nevertheless, CA19-9 levels are possibly valuable in combination with other markers, for example CEA [118–120].

### Mucins

Members of the mucin family are either present as secreted or as transmembrane proteins. Both forms are believed to be involved in inflammation and cancer [121]. When it comes to peritoneal spread, Mucin 16 (MUC16) is considered the most important member of this family. In vitro and in vivo studies suggested that cancer cell adhesion to the peritoneum partly relies on the interaction between MUC16 on ovarian cancer cells and mesothelin on mesothelial cells [122–126]. This interaction is probably mediated by the N-linked oligosaccharides of MUC16. Theoretically, blocking these oligosaccharides with lectins is an attractive therapeutic option [123]. In diagnosing PM, preoperative MUC16 serum levels in gastric cancer patients showed sensitivities ranging from 38.6 to 55 % and specificities between 93.9 and 100 % [113–115, 127, 128]. However, the prognostic value of MUC16 remains inconclusive [80, 127–129].

MUC1 is another mucin described in PM and is expressed on cancer cells [130–133]. It is questionable as to whether this mucin has a role in the attachment phase, since it does not bind mesothelin [122]. Accordingly, the role of MUC1 in clinical settings is so far not convincing [133, 134].

### Epithelial cell adhesion molecule (EPCAM)

EpCAM is a homotypic calcium independent cell adhesion molecule not belonging to one of the previously mentioned groups of molecules [135]. Its expression on cancer cells [98] and its upregulation in PM from gastric cancer [136] suggest a function for this molecule in PM. Its role as adhesion molecule in PM, however, was not confirmed by in vivo antibody experiments in ovarian cancer [98].

In contrast, studies on the therapeutic value of EpCAM were promising, indicating that this molecule might promote peritoneal dissemination through other functions. This is illustrated by treatment with the bispecific antibody anti-EpCAM × anti-CD3 that eradicated PM from ovarian cancer in mice by reactivating tumour-resident T-cells [137]. The bispecific (anti-EpCAM x anti-CD3) trifunctional antibody Catumaxomab was investigated as monotherapy in a phase I/II study, in which this compound was shown to be relatively safe and possibly effective in gastric, colorectal and pancreatic cancer [138]. Concerning its possible diagnostic and prognostic value, data on EpCAM is inconsistent [103, 139].

### Other molecules of interest

Several less frequently studied molecules possibly contribute to tumour cell adhesion as well. These are chemokine receptors, transforming growth factor beta induced gene-h3 (beta ig-h3) and urokinase receptor (uPAR). Although literature on the molecules described in this section suggest that they contribute to cancer cell adhesion to the peritoneum, further research should confirm this assumption.

The chemokine (C-X3-C motif) receptor 1 (CX3CR1) is expressed by ovarian cancer cells and was shown to mediate in vitro tumour cell adhesion to mesothelial cells by interacting with mesothelial chemokine (C-X3-C motif) ligand 1 (CX3CL1) [140]. Expression of another chemokine, chemokine (C-X-C) motif receptor 4 (CXCR4), is expressed on both mesothelial and cancer cells and correlates to worse survival rates in ovarian cancer patients. In vitro and in vivo blocking of CXCR4 with its antagonist ADM3100 was thereby shown to inhibit PM formation [141].

uPAR might also be relevant in PM formation and is detected at the interaction sites of ovarian carcinoma cells and mesothelial cells. In vitro experiments indicated that uPAR mediates tumour cell adhesion by interacting with mesothelial vitronectin [55].

Lastly, beta ig-h3 is an adhesion molecule expressed on mesothelial cells. Upregulation is associated with increased in vitro gastric cancer cell adhesion and the presence of PM [142], suggesting a role for this molecule in PM. Furthermore, in an in vitro ovarian cancer model, peritoneal cells—but not tumour cells—showed high beta ig-h3 levels. This molecule thereby significantly increased ovarian cancer cell adhesion to peritoneal cells, which could be blocked with a beta ig-h3 neutralising antibody [143].

## Discussion

**Fig. 4:**
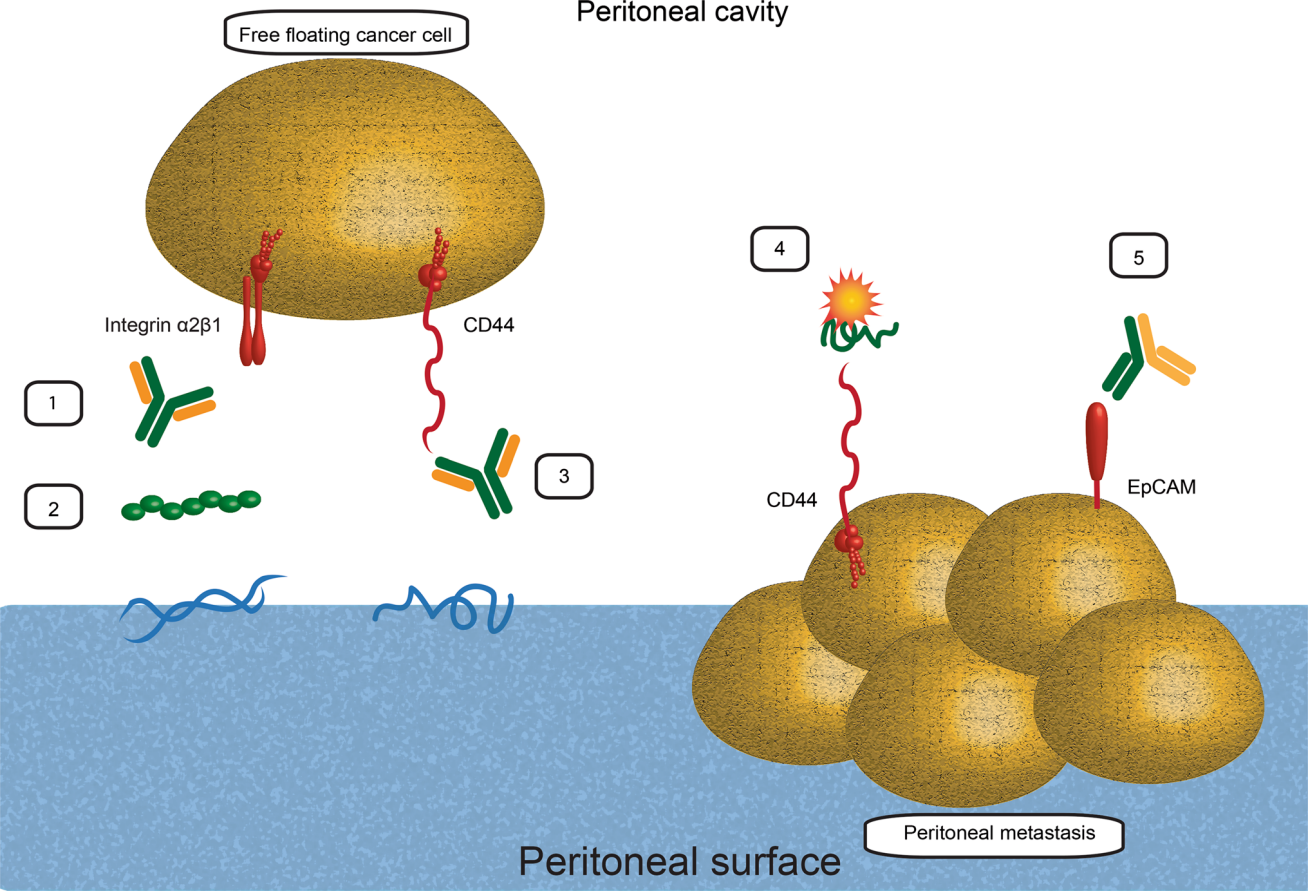
Most promising therapeutic options in prevention (*left*) and treatment (*right*) of peritoneal dissemination: *1* anti-integrin α2β1 antibodies; *2* peptide sequences; *3* anti-CD44 antibodies; *4* hyaluronan bound to cytostatic agents; *5* catumaxomab, a trifunctional antibody with binding sites for EpCAM, T-cells and accessory cells. (Color figure online)

The present study was designed to identify molecules from literature that mediate tumour cell adhesion to the peritoneum and to evaluate their roles in diagnosis, prognosis and therapy of PM. Targeting adhesion molecules may not only prevent tumour cell adhesion and eventually tumour outgrowth in patients at high risk for peritoneal dissemination but the expression of adhesion molecules on tumour cells also allows us to use therapies targeting adhesion molecules in existing peritoneal carcinomatosis (Table 2; Fig. 4). Hence, advancing studies on the therapeutic and diagnostic value of adhesion molecules seems a very promising and rational way for optimising and personalising treatment of patients presenting with peritoneally metastasised CRC.

**Table 2:** Summary of targets with possible clinical implication in PM of colorectal, ovarian, gastric and pancreatic cancer and PMP

Target in PM	Prognostic relevance	Diagnostic relevance	Possible therapeutic implications
Integrins	Yes	Not clear	Promising
	Higher expression of αvβ3 correlated to worse prognosis [30]		Antibodies against integrin α2 and β1 and ECM components [26–28, 30, 31, 33–47]
			Peptide sequences of ECM components [26, 57, 60]
			NF-κB inhibitor (DHMEQ) [48]
			Phospholipids [49]
			Adriamycin bound to SWKLPPS, intraperitoneal [61]
CD44	Yes	Dubious [76, 78, 79]	Promising
	Higher CD44 s expression correlated to worse survival [77, 78]		Antibodies against CD44 and CD44 s [28, 30, 44, 60, 64–70]
			Hyaluronidase, intraperitoneal [28, 34, 64, 65]
			Adhesion barriers [82, 87–89]
			Cisplatin [84], paclitaxel [85] or campthotecin [86] bound to hyaluronan
VCAM1	Not clear	Not clear	Dubious, blocking VCAM1 leads theoretically to less PM [42, 71]
ICAM1	Not clear	Not clear	Dubious, blocking ICAM1 leads theoretically to less PM [42, 69, 73, 94, 96]
L1CAM	Dubious [100]	No [100]	Highly experimental
			Antibodies [98]
			^67^Cu-radiotherapy combined with antibodies, intraperitoneal [101]
Blood group antigens	Yes	Yes	Highly experimental
	CA19-9 levels in serum and peritoneal fluid [80, 103, 104, 108, 109, 111, 112, 116]	CA19-9 levels in serum and peritoneal fluid [103, 104, 108, 109, 113, 114, 117–120]	Antibodies against Le^x^ [105]
			Blocking FUT3 [107]
MUC16	Dubious [80, 127–129]	Yes	Highly experimental
		MUC16 levels in serum and peritoneal lavage [113–115, 127, 128]	Antibodies [124]
		MUC1 PT-PCR [132]	
			Blocking mesothelin [122, 123, 125]
			Anti-MUC1 antibody (C595) combined with docetaxel [130]
EpCAM	Dubious [103, 140]	No [103, 140]	Promising
			Bispecific antibodies EpCAM/CD3 [137]
			Catuxomab monotherapy, intraperitoneal [138]
Chemokine receptors	Not clear	Not clear	Highly experimental
			Antibodies against CX3CR1 and CX3CL1 [140]
			ADM3100 [141]
uPAR	Not clear	Not clear	Highly experimental
			Antibodies [55]
Beta ig-h3	Not clear	Not clear	Highly experimental
			Antibodies [143]

The value of the adhesion molecules is regarded dubious when data on these molecules are severely contradictory or sufficient adequate data is lacking

In PM formation, the roles of CD44 s, integrin α2β1 and MUC16 appeared to be well investigated. Interestingly, integrin α2β1 is not the typical integrin that binds to ECM components. There might be several explanations for this discrepancy. First, according to the available literature tumour cells show upregulation of mainly the α2β1 subunits, meaning that the overall expression profile of integrin subunits might be different from the profile expressed by non-cancer cells. Consequently, interactions observed between tumour cells and mesothelial cells might differ as well. Secondly, as described in the result sections, literature on several other subunits only concerns their roles in vitro. Their roles in vivo, therefore, remain unclear. Due to their in vitro and ex vivo adhesive functions, L1CAM, proteoglycans, betaig-H3 and uPAR might contribute to peritoneal dissemination as well. However, their exact functions and clinical possibilities have to be elucidated. Accordingly, in vitro, ex vivo and in vivo antibody experiments should be developed to assess their adhesive potential. Furthermore, while two systematic reviews support our findings on most adhesion molecules [16, 59], most literature regarding the involvement of adhesion molecules in PM yields contradictory findings. This may be related to heterogeneity of published methods and varying sample sizes. In diagnosis and prognosis of PM, detection of MUC16 and blood group antigens might be useful. Prior to clinical implementation, however, extensive validation of these molecules is necessary. Validation in well-defined patient cohorts is also required for EpCAM, integrin α2β1 and CD44, molecules that have emerged as possibly useful therapeutic targets (Table 2; Fig. 4). Remarkably, while EpCAM showed therapeutic significance in ex vivo and in vivo experiments, its role in in vitro adhesion to the peritoneum was not confirmed. This discrepancy might be attributable to the finding that EpCAM carries out multiple functions, including cell adhesion, cellular signaling, migration, proliferation and differentiation [135, 144–146]. As such, the combination of these mechanisms, as opposed to only a single function (i.e. adhesion), might be of greater importance in promoting PM.

The role of adhesion in haematogenous metastases has been described in several literature studies. Bird et al. (2006) [2] focused on the development of liver metastases from CRC. In both haematogenous spread and spread across the peritoneal cavity—i.e. transcoelomic spread, cancer cells first must detach from the primary tumour to enter the circulation or the peritoneal cavity respectively. Cancer cells, carried by the blood stream or floating in the peritoneal cavity must evade immune defences in order to reach their host organ. At the site of the host organ, adhesive interactions between the organ and cancer cells are required for the development of a metastasis [2, 16, 59]. To disseminate to the liver, tumour cells have to adhere to endothelial cells lining the hepatic sinusoids. Interactions between tumour cells and endothelial cells that are thought to be important for liver dissemination consist of CD44 binding to hyaluronan, the blood group antigens sLea and sLex binding to selectins and mucins binding to ECM molecules [2]. This review, however, did not identify blood group antigens and E-selectin to be important in peritoneal dissemination. Additionally, L1CAM, proteoglycans, betaig-H3 and uPAR might contribute to PM formation, although these molecules were not described in the formation of liver metastases. Thus, we propose that haematogenous and transcoelomic spread differ in respect to several adhesion molecules. So far, no literature has described the exact differences between the mechanisms resulting in liver metastases from CRC and PM from CRC. Difference in adhesion mechanisms can be assumed, since cancer cells have to attach to different kind of cells: to mesothelial cells in peritoneal dissemination and to endothelial cells in hepatic spread. These different cells may express different molecules, making different cell–cell interactions necessary for adhesion. Expression of molecules depends on signalling molecules present in the environment, and thus may differ between the peritoneal surface and the hepatic sinusoids. For example, one study showed insulin-like growth factor 1 (IGF-1) and hypoxia-inducible factor 1-alpha (HIF-1α) to be exclusively overexpressed in PM and not in liver metastases [147]. Difference in growth factors and angiogenic factors might induce different expression patterns in endothelial and mesothelial cells.

Several studies stress the importance of new molecular targets to improve therapy and selection of patients with PM of CRC [8, 13, 14]. The adhesion molecules EpCAM, α2β1 and CD44 s were seen to mediate tumour cell adhesion to the peritoneum and might be particularly useful in the prevention of minimal residual disease in high-risk patients, such as patients with T4 colon tumours [21, 22]. In addition, blocking tumour cell adhesion in the perioperative period may be effective in preventing peritoneal dissemination [23]. A preventive HIPEC procedure might possibly be of additional value in high stage CRC [13]. With respect to a more personalised approach, blocking specific interactions between the mesothelial lining and tumour cell could be of even greater benefit in patients at high risk of peritoneal tumour spread. After blocking interactions between the peritoneal surface and tumour cells, tumours cells may die because of anoikis [16]. Furthermore, most tumour cells circulating in the peritoneal cavity are rapidly removed by the immune system [23]. Accordingly, once adhesion to the mesothelial lining is blocked, the tumour cell may be removed by the body’s own defence mechanisms [23]. This is supported by the observation that the presence of free-floating tumour cells in the peritoneal cavity does not necessarily lead to PM [18, 19].

This extensive assessment of available literature reveals that knowledge on metastasis-specific genes and their possible clinical implications is far from complete. An ‘–omics’ approach, synchronously assessing multiple biomarkers, might help to identify more biomarker candidates since it enables discovery-based research. Ideally, the first step in identifying new biomarker candidates would be the use of mass spectrometry-based proteomics in ex vivo models. In this way, protein expression on both CRC cell lines and patient derived peritoneum can be assessed, enabling comparison of molecules expressed on cancer cells and mesothelial cells. Next, the same proteomic approach in adhesion assays should assess the specific molecules required for adhesion, a process that could be visualised using green fluorescent protein. The previously described steps should be repeated in an environment reminiscing a surgery-induced environment by addition of interleukins. In this way, several possible candidates can be identified that mediate tumour-mesothelial adhesion in both a surgical and non-surgical setting. These candidates should be further studied using antibody blocking in functional assays and animal models. Prior to clinical implication, potential diagnostic, prognostic and therapeutic value of the identified markers should be validated in well-defined patient cohorts. Further studies should reduce the risk of bias associated with evaluation of molecular markers, for example by minimising differences in sample handling. It is thereby important to increase the reproducibility of individual studies using a split-sample for independent validation [148]. Ultimately, increasing reproducibility of genome-wide studies and extensive validation of possible biomarkers could lead to major advances in our understanding of metastasis-specific genes and their clinical possibilities. For CRC patients with PM, the gained knowledge on the diagnostic and therapeutic options of biomarkers will potentially lead to earlier diagnosis and a more personalised, or even preventive, approach and ultimately to better outcomes.

## Abbreviations

CRC: Colorectal carcinoma
PM: Peritoneal metastases
PMP: Pseudomyxoma peritonei
ECM: Extracellular matrix
uPA: Urokinase plasminogen activator
MDR1: Multidrug resistance 1 polypeptide
MRP2: Multidrug resistance protein 2
ICAM1: Intercellular adhesion molecule
VCAM1: Vascular cell adhesion molecule
L1CAM: L1 cell adhesion molecule
NRP1: Neuropilin 1
sLe^a^: Sialyl Lewis a
Le^x^: Lewis x
sLe^x^: Sialyl Lewis x
MUC16: Mucin 16
MUC1: Mucin 1
EpCAM: Epithelial cell adhesion molecule
CXCR4: Chemokine (C-X-C) motif receptor 4
uPAR: Urokinase receptor
Beta ig-h3: Beta induced gene-h3
CX3CL1: Chemokine (C-X3-C motif) ligand 1
IGF-1: Insulin-like growth factor 1
HIF-1α: Hypoxia-inducible factor 1-alpha
